# Mixed Neuroendocrine—Non‐Neuroendocrine Neoplasm (MiNEN) of the Gallbladder. A Case Report From a Low‐Resource Setting

**DOI:** 10.1002/ccr3.73652

**Published:** 2026-09-29

**Authors:** Muhammad Farhan, Muhammad Imran, Beenish Sabir, Khalil Ahmad, Farheen Khan, Shahid Hussain, Zohaib Ahmed, Muhammad Asim, Iqra Mehmood, Rahmat Gul Omarzai

**Affiliations:** ^1^ Department of General Surgery Shaheed Saif Ur Rehman Government Teaching Hospital Gilgit Pakistan; ^2^ Rawalpindi Medical University Rawalpindi Pakistan; ^3^ Department of Histopathology Shifa International Hospital Islamabad Pakistan; ^4^ Department of Medicine Nangarhar University Daronta, Nangarhar Afghanistan

**Keywords:** biliary tract neoplasms, gallbladder neoplasms, immunohistochemistry, mitotic index, neuroendocrine tumors

## Abstract

Mixed neuroendocrine–non‐neuroendocrine neoplasms (MiNENs) of the gallbladder are exceedingly rare entities. We report a case of a 35‐year‐old female with a palpable right hypochondrial mass. Imaging demonstrated a large polypoid gallbladder lesion with periportal lymphadenopathy. The patient underwent open cholecystectomy with lymphadenectomy. Histopathology and immunohistochemistry revealed a MiNEN. At 12‐month follow‐up, she remained free of recurrence without any adjuvant chemotherapy.

## Key Clinical Message

Mixed neuroendocrine–non‐neuroendocrine neoplasms (MiNENs) of the gallbladder are exceptionally rare and diagnostically challenging. This case highlights the importance of thorough histopathological and immunohistochemical evaluation for accurate diagnosis, as well as the need for individualized treatment strategies given the absence of standardized management protocols for such tumors.

## 1 Introduction

Malignant neoplasms of the gallbladder are rare, accounting for approximately 0.5% of all cancer diagnoses. While adenocarcinoma is the most common histological subtype, typically found in the fundus, other less frequent types include small cell carcinoma and acinic cell carcinoma [1]. Mixed Neuroendocrine–Non‐Neuroendocrine Neoplasm (MiNENs) represents an uncommon variant, defined in 2017 as a tumor with distinct neuroendocrine and non‐neuroendocrine components, each comprising at least 30% of the tumor mass. The non‐neuroendocrine portion is most often adenocarcinoma, though it can also manifest as squamous cell carcinoma or acinic cell carcinoma [2]. MiNENs arising in the biliary tract constitute a small fraction, specifically 4%–24%, of all digestive MiNENs [3]. The inherent heterogeneity of MiNENs complicates the development of standardized diagnostic criteria and treatment protocols, necessitating personalized patient management and posing significant challenges. This report presents a case of gallbladder MiNEN in a young female patient, detailing her diagnostic journey, treatment, and prognosis.

## 2 Case History and Examination

A 35‐year‐old female presented to the General Surgery Outpatient Department of our hospital with complaints of right hypochondrial and epigastric pain, nausea, and acid brash for the past month, occurring on and off. The pain was aggravated by food intake, especially fatty foods and milk products, and was relieved only by oral analgesics and proton pump inhibitors; at times, intravenous analgesics were required. She had previously been taking medications from a dispensary without undergoing any proper investigations. She is normotensive and non‐diabetic.

This time, on presentation to our OPD, a general physical examination and abdominal examination were performed, which revealed tenderness in the right hypochondrium, along with a palpable globular mass. The patient was pale and emaciated.

## 3 Biochemical and Radiological Investigations and Treatment

She was admitted to the female surgical ward and advised baseline investigations (CBC, LFTs, RFTs, S/E, serum amylase, blood grouping and cross‐match, and viral serology for Hepatitis B & C), along with ECG, chest X‐ray, and ultrasonography (USG) of the abdomen with hepatobiliary focus. Biochemical investigation reports are shown in Table 1.

**TABLE 1:** Biochemical parameters reported at initial investigations.

Biochemical parameters	Values
Hb	10.2 g/dL
Platelets	212 × 10^9^ /L
TLC	9,100 cells/μL
MCV	85 fl
Total Bilirubin	0.9 mg/dL
ALT	36 U/L
ALP	116 U/L
Creatinine	0.7 mg/dL
Urea	26 mg/dL
CA19‐9	13.3 U/mL

USG abdomen reported a large, polypoidal, broad‐based, heterogeneous mass lesion bulging into the gallbladder lumen from the anterior wall. Color Doppler imaging showed vascularity within the lesion. These features suggested carcinoma of the gallbladder. Serum CA 19–9 and CEA were ordered. A triphasic contrast‐enhanced CT (oral and IV contrast) of the abdomen was performed, which revealed a well‐defined, irregularly shaped, lobulated, polypoidal enhancing mass extending into the lumen from the posteromedial wall of the gallbladder, with enlarged periportal lymph nodes as shown in Figure 1.

**FIGURE 1:**
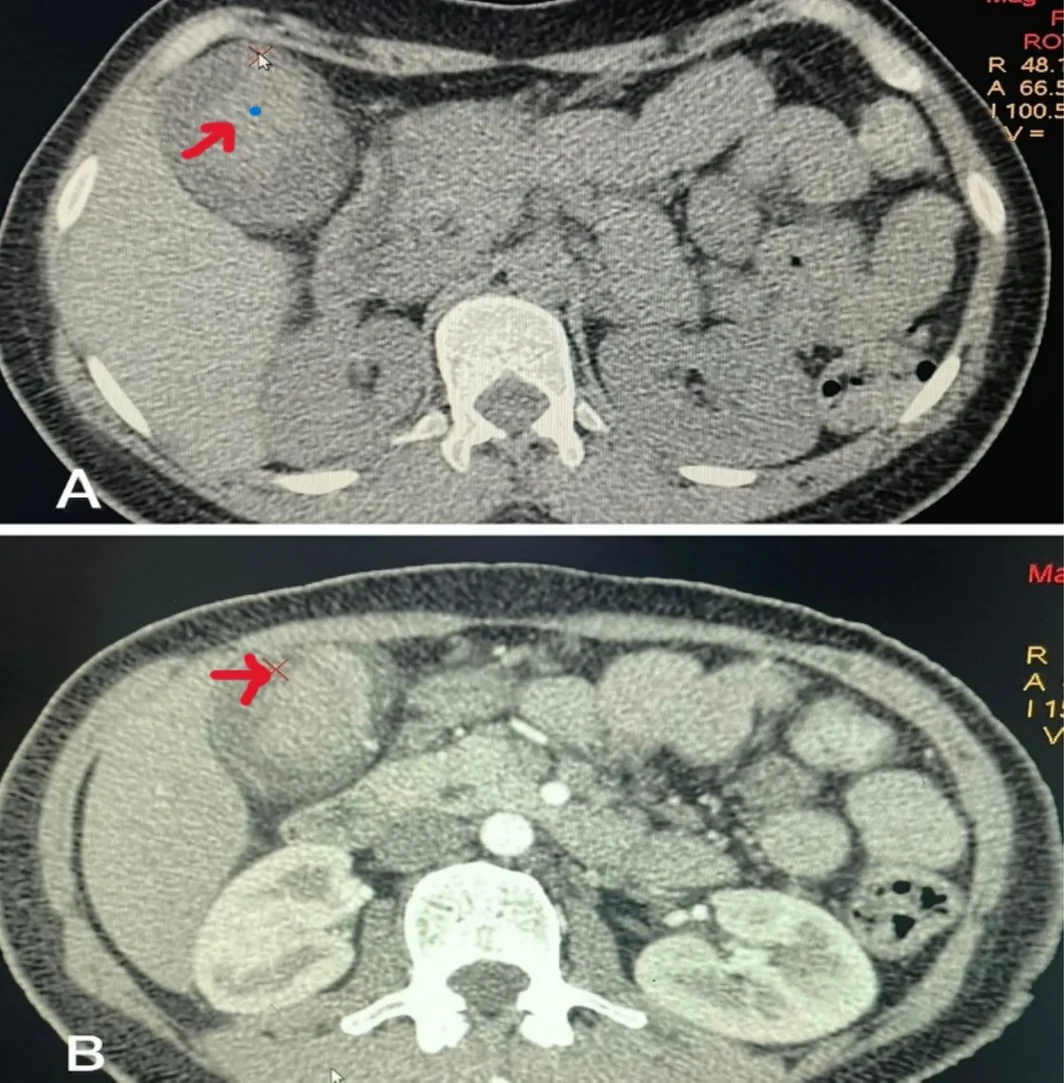
Triphasic CT Scan Abdomen with IV and Oral Contrast. (A) Plain‐phase axial image showing a well‐defined lobulated mass arising from the GB wall (red arrow). (B) Arterial phase contrast‐enhanced axial image demonstrating heterogeneous enhancement of the GB mass extending into the lumen and abutting the hepatic surface.

The case was discussed in the tumor board meeting, which advised complete surgical excision or image‐guided biopsy if the mass was inoperable. Considering the patient's financial constraints, as she could not afford an image‐guided biopsy in a private setup, we decided to proceed with complete surgical excision. After optimizing the patient, an open cholecystectomy under general anesthesia was performed. The liver was examined intraoperatively, and no lesion was found. However, there was extensive lymphovascular invasion. A drain was placed in the subhepatic space, which was removed on the second postoperative day as the output was nil. Postoperative recovery was uneventful.

The entire specimen along with resected lymph nodes was sent for histopathology, which revealed a 3.5 × 2.5 × 1.0 cm white friable tumor in the wall of the gallbladder body with perineural and lymphovascular invasion. Histopathology reported the lesion as a Mixed Neuroendocrine–Non‐Neuroendocrine Neoplasm (MiNEN), with small‐cell neuroendocrine carcinoma comprising 70% and moderately differentiated adenocarcinoma 30%, as shown in Figure 2. The pathological stage was pT2bN1, and all margins were negative.

**FIGURE 2:**
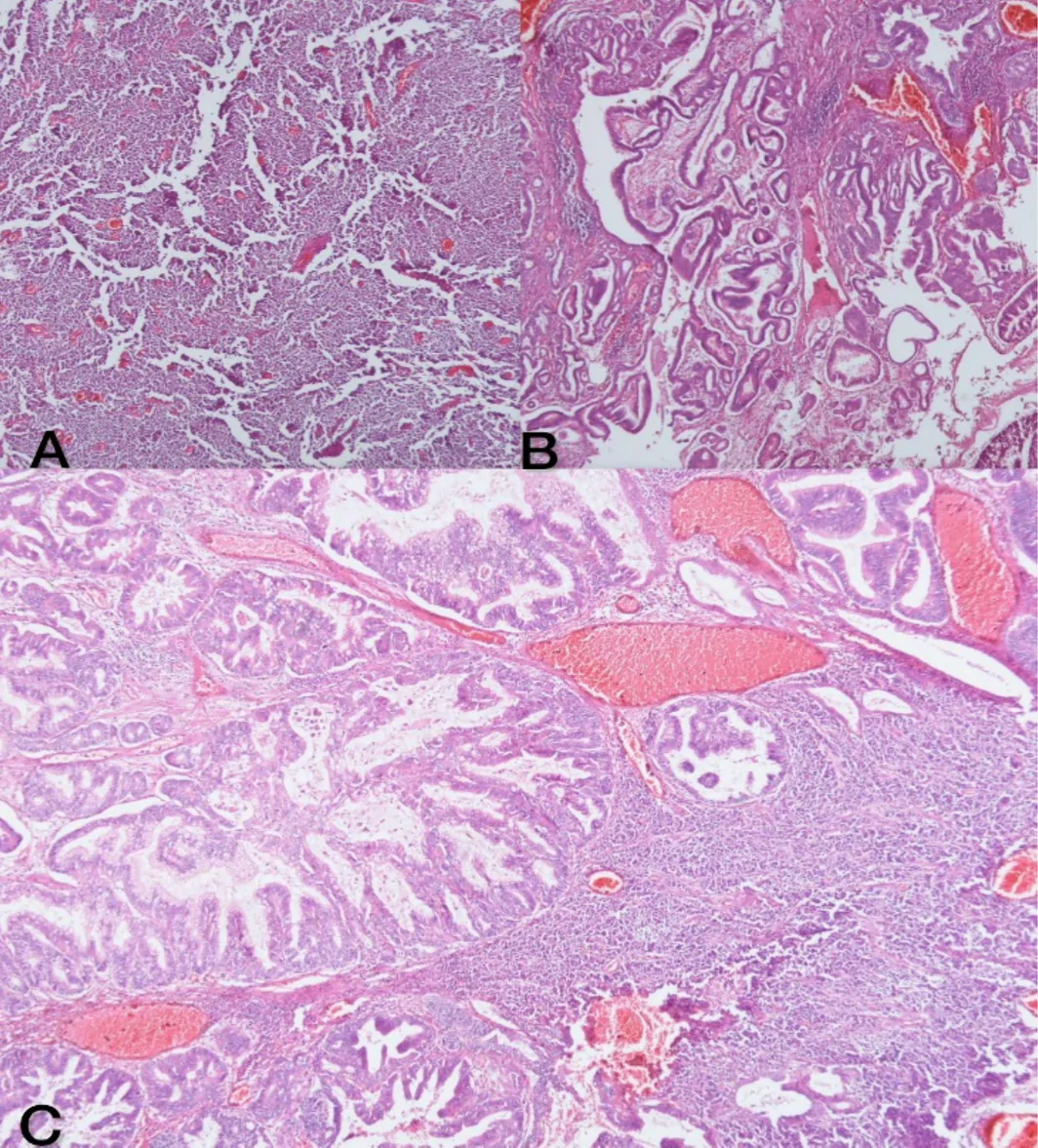
Histopathological Evaluation of Specimen. (A) Sheet and nested configuration of small round blue cells at 4 × (Small Cell Neuroendocrine Component), (B) Well‐formed glandular configuration of tumor at 4 × (Adenocarcinoma Component), (C) This photomicrograph highlights both small cell NE (bottom right) and adenocarcinoma (top left) components.

Immunohistochemistry showed Synaptophysin and Chromogranin positivity in the small‐cell carcinoma, with dot‐like positivity for CAM 5.2 and CK AE1/AE3 as shown in Figure 3. The adenocarcinoma component showed positivity for CK19 and CDX2, as shown in Figure 3.

**FIGURE 3:**
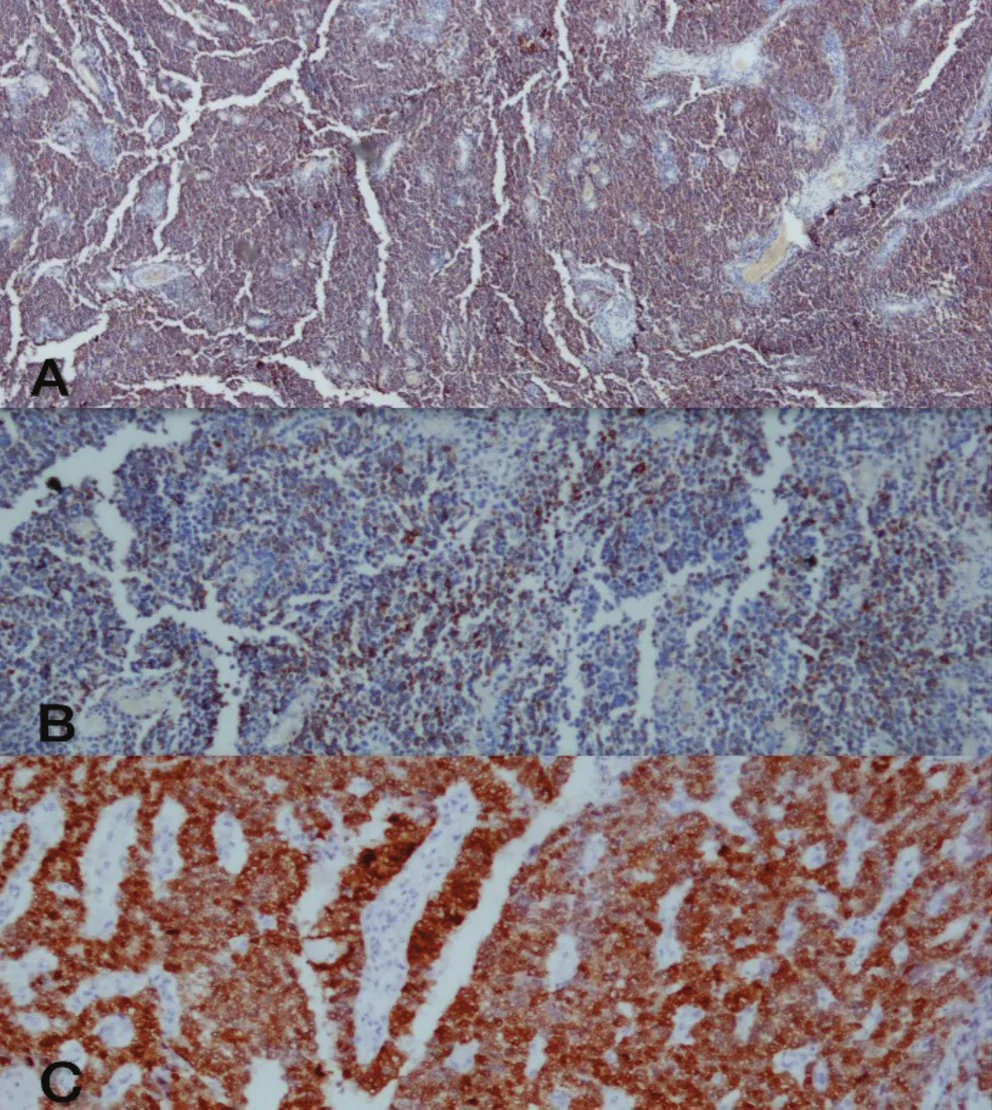
Immunohistochemical Evaluation of Specimen. Photomicrograph (A) shows Synaptophysin positivity, (B) Shows Chromogranin positivity in the small Cell NE component, while (C) shows positivity for CDX2 in the adenocarcinoma component.

## 4 Results and Follow‐Up

The patient remained stable postoperatively and was discharged on the 6th postoperative day. She was referred to an oncology center for adjuvant chemotherapy but declined due to financial limitations. On follow‐up 12 months later, there were no clinical or radiological signs of recurrence.

## 5 Discussion

MiNENs are a rare and complex group of tumors that combine neuroendocrine and non‐neuroendocrine components [4]. This duality often leads to diagnostic and therapeutic challenges due to the heterogeneous nature of the tumors. Gallbladder MiNENs are particularly rare, and their pathogenesis, clinical features, and management remain poorly understood [5]. Despite the limited number of published cases, several studies have attempted to elucidate the presentation, diagnosis, and management of these tumors.

The most common histopathologic subtype of MiNEN in the gallbladder is mixed adenocarcinoma and neuroendocrine carcinoma. The neuroendocrine component in these cases typically presents with cells showing high‐grade atypia, frequent mitotic figures, and prominent neuroendocrine markers, while the adenocarcinomatous portion often features glandular differentiation [6]. In our case, the histopathology report revealed a similar combination of components, with the neuroendocrine component (small cell neuroendocrine carcinoma, SCNEC) contributing 70% and the non‐neuroendocrine (moderately differentiated adenocarcinoma) component 30%. In a case published by Skalicky et al. a 56‐year‐old woman was diagnosed with MiNEN of the gallbladder with histopathological predominance of the neuroendocrine component over the non‐neuroendocrine component [7]. Moreover, Ren et al. have reported a case of a 70‐year‐old female with a polypoid mass in the neck and body of the gallbladder. The histopathology revealed 65% LCNEC and 35% moderately differentiated papillary adenocarcinoma, with a distinct transitional zone between the two components [5]. Meanwhile, Kadota et al. have reported an unusual case of a 70‐year‐old Japanese woman with well‐to‐moderately differentiated adenocarcinoma with a focal neuroendocrine component (< 30%) positive for synaptophysin, chromogranin A, and CD56, and a Ki‐67 index > 80%. The tumor was diagnosed as gallbladder adenocarcinoma with neuroendocrine differentiation because it did not meet the criteria for MiNEN [8].

Immunohistochemistry in MiNENs is crucial for confirming the neuroendocrine nature of the tumor. Common markers include chromogranin A, synaptophysin, and CD56. Our case revealed strong expression of synaptophysin and chromogranin, and dot‐like positivity of Cam5.2, which supports the diagnosis of the neuroendocrine component. This finding is consistent with other published cases [5, 8]. Also, the adenocarcinomatous component of MiNEN in our case showed glandular differentiation similar to findings in case reports by Skalicky et al. [7] and Ines et al. [9]. Another important aspect in the classification and identification of MiNENs is the Ki‐67 proliferation index and molecular clustering. Based on the Ki‐67 index of the neuroendocrine (NE) component, neuroendocrine tumors (NETs) are classified into three grades: Grade 1, with a Ki‐67 index of < 3%; Grade 2, with an index of 3%–20%; and Grade 3, with an index of > 20%. In contrast, neuroendocrine carcinomas (NECs) typically demonstrate a Ki‐67 proliferation index of > 60% [4, 6, 10]. In MiNENs, the average Ki‐67 index of the NEC component has been reported to be approximately 70%. In our specimen, the small‐cell carcinoma component demonstrated a Ki‐67 proliferation index of 90%, which is highly consistent with the findings reported in the literature.

Extrapulmonary neuroendocrine carcinomas (EPNECs) have also been classified at the molecular level based on their gene‐expression profiles. The first molecular cluster is characterized by high INSM1 expression, with variable expression of ASCL1/DLL3 or NEUROD1, whereas the second cluster predominantly demonstrates overexpression of YAP1 and NOTCH1 [11]. Some authors have proposed that MiNENs may develop through transdifferentiation of a non‐neuroendocrine tumor into a neuroendocrine phenotype, potentially driven by alterations involving C‐MYC and SMARCA mutations [4]. Unfortunately, molecular & genetic profiling could not be performed in our case because these facilities are not currently available in our setting.

The use of imaging modalities such as CT scans and MRIs can be quite useful in detecting tumors, although the mixed nature of these tumors may make them difficult to characterize on imaging alone. Our case involved a triphasic CT scan, revealing a well‐defined mass with features suggestive of gallbladder carcinoma, but with limited information on the nature of the tumor. These imaging findings are consistent with published literature, where uncertainty remained regarding the nature of the tumor [5, 12, 13]. Tsuchiyose et al. have reported the need for endoscopic ultrasound‐guided diagnosis of MiNEN of the gallbladder with anomalous pancreaticobiliary junction in a 46‐year‐old female [14]. However, in a case report published by Seki et al. the diagnosis of gallbladder MiNEN was established after surgery for acute cholecystitis [15]. So, only endoscopic or imaging‐guided biopsy can help to establish a diagnosis pre‐operatively.

The most common approach to MiNENs of the gallbladder involves surgical resection, which is considered the most effective treatment for localized disease. In our case, the patient underwent an open cholecystectomy with lymphadenectomy, which is consistent with standard care for gallbladder malignancies. This complies with the case series and literature review by Zhang et al. in which radical cholecystectomy was done regardless of tumor stage and grade, followed by favorable patient outcomes [16]. Several other case reports have been published in which primary treatment for gallbladder MiNENs remained surgical resection [5, 14].

Chemotherapy regimens often include agents such as Cisplatin and Etoposide, Gemcitabine, and Durvalumab, which have been effective in treating neuroendocrine tumors in other parts of the body. However, the response to these regimens can be unpredictable in MiNENs [17, 18]. They have reported the efficacy of immunotherapy, PD‐1 inhibitors, in patients with advanced metastatic disease with positive expression of PD‐L1 receptors [18]. Inconsistency in response to chemotherapeutic agents in advanced metastatic MiNENs is also reported by Angelico et al. [19] and Oe et al. [20].

For gallbladder mixed neuroendocrine–non‐neuroendocrine neoplasms (MiNENs), the prognosis can be highly variable. It is influenced by the disease stage at diagnosis, the specific histological components, and the treatment approach. Poor prognosis in terms of overall survival or recurrence has been reported by Ren et al. and Park et al. [5, 13]. While Seki et al. have reported favorable clinical outcomes in which the patient remained recurrence‐free for 36 months after surgical resection of the tumor without adjuvant therapy [15]. Favorable clinical outcomes and good prognosis in patients with gallbladder MiNENs, after surgical and adjuvant therapies, have been documented in the literature [18, 20, 21].

MiNENs of the gallbladder pose diagnostic, treatment, and prognostic challenges due to their rarity and complex histological composition. Diagnosis requires a combination of imaging, histopathology, and immunohistochemistry to identify both neuroendocrine and non‐neuroendocrine elements. Treatment protocols are not standardized, with surgery as the primary approach, while the role of adjuvant therapies remains uncertain. Prognosis is generally poor, although a predominance of the neuroendocrine component may suggest a better outcome. Future research should focus on developing standardized treatment protocols, identifying biomarkers for treatment response, and exploring targeted therapies to improve patient outcomes.

## Author Contributions

**Muhammad Farhan:** conceptualization, methodology, writing – original draft, data curation. **Muhammad Imran:** data curation, supervision, writing – review and editing, writing – original draft. **Beenish Sabir:** writing – original draft, investigation, methodology. **Khalil Ahmad:** data curation, writing – review and editing. **Farheen Khan:** writing – review and editing, investigation. **Shahid Hussain:** writing – review and editing, supervision. **Zohaib Ahmed:** writing – review and editing, supervision. **Muhammad Asim:** writing – review and editing, supervision. **Iqra Mehmood:** writing – review and editing, supervision, resources. **Rahmat Gul Omarzai:** writing – review and editing, supervision.

## Funding

The authors have nothing to report.

## Ethics Statement

Written consent for publication has been obtained from the patient described in this case report, and ethical approval was not required for this study in accordance with local or national guidelines.

## Consent

Written informed consent was obtained from the patient described in this case report for publication of the medical case details and any accompanying images. Written informed consent was obtained from the patient to publish this report in accordance with the journal's patient consent policy. Ethical approval was not required for this study in accordance with local or national guidelines.

## Conflicts of Interest

The authors declare no conflicts of interest.

## Data Availability

The data that support the findings of this study are available from the corresponding author upon reasonable request.
